# Is serum level of CC chemokine ligand 18 a biomarker for the prediction of radiation induced lung toxicity (RILT)?

**DOI:** 10.1371/journal.pone.0185350

**Published:** 2017-09-28

**Authors:** Eleni Gkika, Werner Vach, Sonja Adebahr, Tanja Schimeck-Jasch, Anton Brenner, Thomas Baptist Brunner, Klaus Kaier, Antje Prasse, Joachim Müller-Quernheim, Anca-Ligia Grosu, Gernot Zissel, Ursula Nestle

**Affiliations:** 1 Department of Radiation Oncology, Medical Center – University Hospital Freiburg, Freiburg, Germany; 2 Institute of Medical Biometry and Statistics, Medical Faculty & Medical Center, University of Freiburg, Freiburg, Germany; 3 German Cancer Consortium (DKTK), partner site Freiburg, Germany; 4 German Cancer Research Center (DKFZ), Heidelberg, Germany; 5 Faculty of Medicine, University of Freiburg, Freiburg, Germany; 6 Department of Pneumology, Hannover Medical School, Hannover, Germany; 7 Department of Pneumology, University Medical Center Freiburg, Freiburg, Germany; Technische Universitat Dresden, GERMANY

## Abstract

The CC chemokine ligand 18 (CCL18) is produced by alveolar macrophages in patients with fibrosing lung disease and its concentration is increased in various fibrotic lung diseases. Furthermore CCL18 is elevated in several malignancies as it is produced by tumor associated macrophages. In this study we aimed to analyze the role of CCL18 as a prognostic biomarker for the development of early radiation induced lung toxicity (RILT), i.e. radiation pneumonitis after thoracic irradiation and its significance in the course of the disease. Sixty seven patients were enrolled prospectively in the study. Patients were treated with irradiation for several thoracic malignancies (lung cancer, esophageal cancer, thymoma), either with conventionally fractionated or hypo-fractionated radiotherapy. The CCL18 serum levels were quantified with ELISA (enzyme-linked immunosorbent assay) at predefined time points: before, during and at the end of treatment as well as in the first and second follow-up. Treatment parameters and functional tests were also correlated with the development of RILT.Fifty three patients were evaluable for this study. Twenty one patients (39%) developed radiologic signs of RILT Grade >1 but only three of them (5.6%) developed clinical symptoms (Grade 2). We could not find any association between the different CCL18 concentrations and a higher incidence of RILT. Statistical significant factors were the planning target volume (odds ratio OR: 1.003, p = 0.010), the volume of the lung receiving > 20 Gy (OR: 1.132 p = 0.004) and age (OR: 0.917, p = 0.008). There was no association between serial CCL18 concentrations with tumor response and overall survival.In our study the dosimetric parameters remained the most potent predictors of RILT. Further studies are needed in order to estimate the role of CCL18 in the development of early RILT.

## Introduction

Radiation therapy is an important cornerstone of the curative treatment of lung and esophageal cancers and plays also a significant role in the palliative setting in terms of symptom control. The lung is one of the most radiosensitive organs. Radiation induced lung toxicities (RILT) range between 5–50% of the patients irradiated for lung cancer [1] and could be a potentially life threatening risk to patients treated for thoracic tumors. In order to reduce the risk of RILT one should reduce the delivered dose and may thus hamper local control. To date only dosimetric parameters such as the mean lung dose (MLD), the volume of the lung receiving more than 20 Gy (V20) or 5 Gy (V5) are used routinely to predict the risk of RILT. [1–3]. However, dosimetric variables tend to be very collinear (i.e., increasing V20 tends to lead to an increase in the other parameters), and therefore differences in prognostic value among different dosimetric variables may be small. [3]

In an attempt to search for biological predictors for RILT, apart from dosimetric parameters, many studies looked at correlations between the risk of pulmonary injury and variations in several profibrogenic and proinflamatory cytokines such as transforming growth factor ß1 (TGF-ß1), tumor necrosis factor-a (TNF-a) interleukin-1(IL-1), IL-6, high-molecular weight mucin-like antigen KL-6, and platelet-derived growth factor -ß (PDGF- ß)[4, 5]. Because these cytokines are thought to be key mediators in lung toxicity many of them were examined as biomarkers for the early detection of pulmonary toxicities but at the present time there are no reliable and validated predictive assays for treatment decision. [4].

The CC chemokine ligand CCL18 is a chemokine produced by human myeloid cells.[6] In general, macrophage activation by T-helper 2 (Th2) cytokines induces a special phenotype in macrophages, termed ‘‘alternative activation” (M2 phenotype).[6–8] Alternatively activated macrophages play a role in tissue repair processes such as wound healing and fibrosis. [7] In patients, whose disease is associated with fibrotic lung remodeling such as in idiopathic interstitial pneumonias, systemic sclerosis and idiopathic pulmonary fibrosis, it is suggested that the M2 phenotype of the alveolar macrophages leads to exaggerated production of the CCL18 and it is suggested that its serum concentrations might serve as a biomarker of pulmonary disease activity in these patients with idiopathic interstitial pneumonias, systemic sclerosis and idiopathic pulmonary fibrosis,.[9–12] In addition, it has been shown that CCL18 fosters fibrotic processes [13] in pulmonary fibrosis which might drive fibrotic remodeling in RILT.

On the other hand a complex network of chemokines influence the development of primary tumors and metastases [14] and it could be possible that circulating chemokines could be useful tumor prognostic markers. [15] It has been previously published that there is an enhanced production of CCL18 in several malignancies such as ovarian cancer, gastric cancer, breast cancer, colorectal cancer and adenocarcinoma of the lung. [16–22]

The present study was designed to evaluate CCL18 as a predictor for early RILT i.e. radiation pneumonitis as defined per NCI Common Terminology Criteria for Adverse Events v4.03. (National Cancer Institute: Common Terminology Criteria for Adverse Events Version 4.03, CTCAE 2010). For the pathogenesis of the radiation injury theory, fibrosis is a result of abnormally healed inflammatory changes during the pneumonitis stage.[5] Our aim was to investigate the prognostic role of CCL18 for radiation inflammation i.e. pneumonitis which leads to permanent injury i.e. fibrotic remodeling of the lung, hypothesizing that an increase of the CCL18 concentrations could be predictive for lung injury after radiotherapy. We also evaluated several other parameters that may affect the incidence of RILT such as treatment and patient specific characteristics. Furthermore, we investigated the role of this chemokine as a tumor response marker, as it has been suggested that CCL18 concentrations correlate with survival time in adenocarcinomas of the lung[22] but also in other malignancies [23]

## Materials and methods

### Study population and treatment characteristics

The study was approved by the ethic committee of the University Medical Center Freiburg. Written informed consent was obtained from all patients who participated in this study.

Consecutive routine patients receiving thoracic radiation therapy regardless of histology or treatment intention were included in this prospective study. Patients with breast cancer were excluded. The majority were treated for lung cancer or esophageal cancer. PET CTs or CTs as part of the initial staging as well as for treatment response were routinely performed every three months. Staging was assessed according to the UICC 7^th^ Edition. Pulmonary function tests were also obtained before treatment. Other parameters tested were age, the presence of chronic obstructive pulmonary disease (COPD), diabetes mellitus, nicotine consumption and medications such as angiotensin-converting-enzyme inhibitors (ACE inhibitors) or prednisone.

Dose volume histogram (DVHs) parameters such as the planning target volume (PTV) or the volume of both lungs receiving more than 20 Gy (V20) were also tested. Furthermore we calculated the biologically effective dose (BED) and the equivalent dose in 2 Gy fractions (EQD2) prescribed to the PTV.

### Serum sampling and enzyme-linked immunosorbent assay (ELISA)

Serial plasma specimens from each individual were obtained before initiation of the radiation therapy, during treatment, at the end of therapy and at the first and second follow-up.

Venous blood samples were taken using a routine procedure. Blood samples rested for 20 minutes before centrifugation. After centrifugation serum samples were frozen at -80°C within 2 hours. CCL18 was quantified using a DuoSet ELISA Development System kit (R&D Systems Europe).

### Follow up and clinical evaluation of toxicity

Patients were evaluated weekly during radiation therapy as well as every two to three months thereafter. During the first and second follow up (median 3 and 7 months after treatment initiation) beside of the blood sampling patients underwent a chest computed tomography scan (CT). Radiation pneumonitis was scored using the National Cancer Institute’s Common Terminology Criteria for Adverse Events v4.0. (National Cancer Institute: Common Terminology Criteria for Adverse Events Version 4.03, CTCAE 2010). Tumor response was assessed according to the RECIST criteria [24]

### Statistical analysis

Statistical analysis was performed for the absolute CCL18 concentrations at each time point, the difference from baseline for every time point, and the slope of a regression line fitted to the first 3 time points in order to measure the steepness of the CCL18 concentration changes. Furthermore we divided the individual CCL18 time courses into stable (less than 20% variance from the baseline), downwards (≤ 20% decrease from baseline), upwards (≥ 20% increase from baseline) as well as their combinations over the first three time points. These variables were then correlated with the presence of RILT. Logistic regression analyses were performed to determine factors predictive for radiation pneumonitis Grade ≥ 1. Results are reported as odds ratios (OR), 95% confidence intervals (CI), and p-values. In order to investigate a potential influence of the occurrence of RILT on subsequent CCL18 concentration changes we compared CCL18 values post RILT with CCL18 values prior to RILT in a linear mixed model with CCL18 concentrations as outcome, the pre/post status as binary covariate, the five time points as categorical covariates, and the individual intercept as random effect. In the main analysis (prognostic value of CCL18 values and their development of time for development of RILT) a Bonferroni correction was applied to adjust for investigating 12 different, potential associations. Overall survival (OS), local tumor control (LC) and progression free survival (PFS) and time until RILT were analyzed with respect to their association with the above mentioned variables by use of the Cox proportional hazard model. Only patients not yet having experienced the corresponding events were included in the analysis, and the time until event was defined as starting with the last time point involved in the definition of a variable. Patients were censored 6 months after the last blood sample or at loss to follow up. In analyzing local tumor control, death was regarded as a censoring event. The statistical significance level was set at 0.05. All *p* values were two-sided. Statistical analysis was performed using SPSS version 23(SPSS, Chicago Il) and STATA version 14.1.

### Endpoints

The endpoint of this study was to evaluate the role of CCL18 as a prognostic biomarker for the development of early RILT. Secondary endpoints were clinical and treatment related factors and their role in predicting RILT. In an additional exploratory analysis the correlation between CCL18 concentrations and overall survival, local control and progression free survival at every given time-point was evaluated.

## Results

### Patient and treatment characteristics and toxicity

From August 2011 to February 2012, 67 patients were prospectively included in the study. Of these patients 14 were excluded because of insufficient data (less than three CCL18 measurements), withdrawal of informed consent or death before reaching a follow up more than 6 months after treatment completion. Patient and treatment characteristics are described in Table 1. Forty-three patients were treated for lung cancer; eight had an esophageal cancer and one a thymoma. Patients were treated either with conventionally fractionated (n = 41) or hypo-fractionated (n = 12) radiotherapy, 8 patients were treated adjuvant, 2 neoadjuvant, 8 with stereotactic body radiotherapy (SBRT), 31 with a concurrent chemoradiotherapy and 4 in palliative intent. IMRT was performed in 70% of the cases with a mean dose of 53 Gy (range 30–76) Gy. The mean V20 was 15% (range 0.45–36). Fourteen patients had a V20 more than 20%. Over a period of 6 months 21 patients (39.6%) presented with radiological signs of pneumonitis Grade ≥ 1; 3 of them (5.6%) developed symptoms (CTC Grade 2 pneumonitis). There were no Grade ≥ 3 toxicities.

**Table 1:** Patient and treatment characteristics.

Variable	Nr. of patients(%)	Median (range)
**Gender**		
Male	34 (64%)	
Female	19 (36%)	
**Age**		65 (30–83)
**Nicotine consumption(present)**		
Yes	43 (81%)	
No	10 (19%)	
**ACE-Inhibitors**		
Yes	8 (15%)	
No	45 (85%)	
**Prednisone**		
Yes	9 (17%)	
No	44 (83%)	
COPD*		
GOLD 0–2	43 (81%)	
GOLD 3–4	10 (19%)	
**Diabetes mellitus**		
Yes	10 (19%)	
No	43 (81%)	
**FEV 1**		
≤2 l	19 (36%)	
> 2 l	19 (36%)	
n.a.	15 (28%)	
**Tumor**		
Lung cancer	44 (83%)	
Esophageal cancer	8 (15%)	
Thymoma	1 (2%)	
**Histology**		
Undifferentiated	3 (6%)	
SCC	20 (37%)	
Adenocarcinoma	24 (45%)	
SCLC	3 (6%)	
Large cell carcinoma	1 (2%)	
Thymoma	1 (2%)	
n.a.	1 (2%)	
**UICC**		
Stage I	5 (9%)	
Stage II	8 (15%)	
Stage III	32 (61%)	
Stage IV	8 (15%)	
**Treatment**		
Neoadjuvant	2 (4%)	
Adjuvant	8 (15%)	
Concurrent CRT	31 (58%)	
SBRT	8 (15%)	
Palliative RT	4 (8%)	
**Treatment delivery**		
IMRT	37 (70%)	
3D	16 (30%)	
**V20**		15 (0.45–36) %
**PTV Volume**		368 (13–1288) ml
**Physical Dose**		53 (30–76) Gy
**EQD2**_**10**_ **to PTV**		58.4 (34–76) Gy

Abbreviations CRT = chemoradiotherapy, RT = radiotherapy, SCC = squamous cell carcinoma, SCLC = small cell carcinoma, IMRT: intensity modulated radiation therapy, V20 = % volume of the lung receiving more than 20 Gy, PTV = planning target volume, EQD2 = equivalent dose in 2 Gy fraction, COPD = chronic obstructive pulmonary disease, ACE- inhibitors = angiotensin-converting-enzyme inhibitors, FEV 1 = Forced expiratory volume in 1 second, n.a. = not available.

*COPD was dichotomized as not significant (COPD GOLD 0–2) and significant (COPD GOLD 3–4).

### Correlation between clinical and treatment factors

Several factors were tested as potential prognostic markers for RILT. Results are shown in Table 2. Prognostic factors for RILT were age (OR 0.917, *p* = 0.008), the volume of the PTV (OR 1.003, *p* = 0.010), the V20 (OR 1.132, *p* = 0.004) and V20>20% (OR: 4.050, *p* = 0,003).

**Table 2:** Univariate analysis of factors prognostic for radiation pneumonitis Grade ≥ 1.

	UVA	
	OR (95% CI)	P value
Age	0.917 (0.861–0.978)	0.008
FEV 1 ≤ 2l	1.905 (0.521–6.962)	0.330
Nicotine	1.680 (0.382–7.395)	0.493
COPD GOLD 0–2	1.680 (0.382–7.395)	0.484
Diabetes mellitus	0.316 (0.060–1.665)	0.144
Prednisone	3.870 (0.846–17.673)	0.081
ACE-Inhibitors	0.456 (0.083–2.512)	0.367
PTV Volume	1.003 (1.001–1.005)	0.010
V20	1.132(1.041–1.231)	0.004
V20>20%	4.050(1.118–14.674)	0.033
IMRT	0.542 (0.165–1.780)	0.313
Dose	1.033 (0.984–1.085)	0.179
BED	1.006 (0.960–1.053)	0.811
EQD2	1.006 (0.953–1.063)	0.824
Adjuvant treatment	1.647 (0.363–7.465)	0.518
Histology	1.034 (0,490–1,896)	0.919
T1	0.175 (0.016–1.881)	0.150
T2	0.437 (0.061–1.881)	0.413
T3	0.436 (0.100–1.916)	0.273
T4	0.729 (0.153–3.474)	0.692
Tumor progression*	0.731 (0.232–2.306)	0.593

Abbreviations: OR = odds ratio, CI: confidence interval, UVA: univariate analysis.

COPD was dichotomized as not significant (COPD GOLD 0–2) and significant (COPD GOLD 3–4).

*Within 6 months after treatment completion.

### Prognostic value of CCL18 for the development of RILT

#### Absolute concentrations

**Fig 1:**
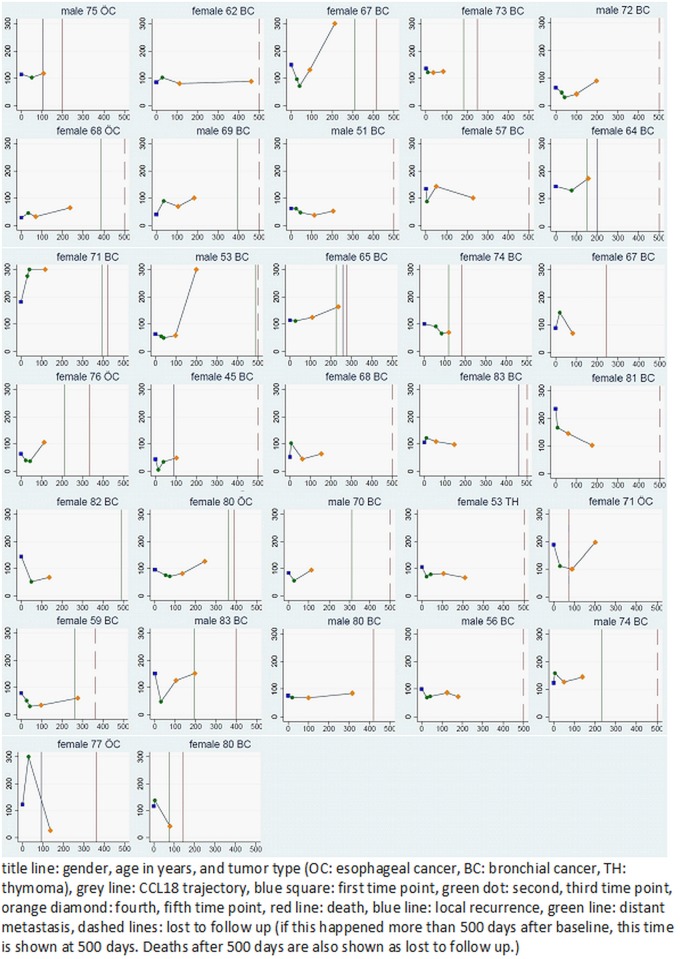
Individual trajectories of CCL18, local recurrence and distant metastases in patients without RILT.

**Fig 2:**
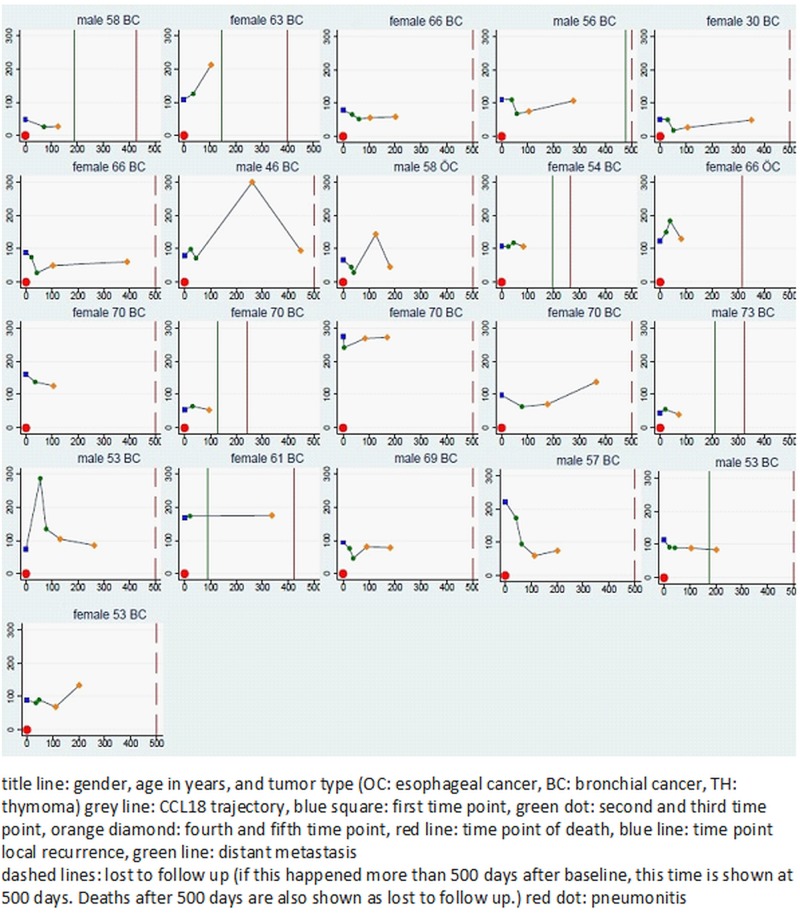
Individual trajectories of CCL18, local recurrence and distant metastases in patients with RILT.

Figs 1 and 2 give an overview about the individual time courses of the CCL18 measurements. The mean CCL18 levels, for the whole group of patients, were 107±50 ng/ml before treatment and 81±77 ng/ml at the end of treatment (S1 Table). During the first (3 months from treatment) and second follow-up (6 months from treatment) the mean CCL18 levels were 93±57 ng/ml and 104±49 ng/ml, respectively. The average slope over the first three time points was -0.348 ng/ml/day for all patients. We could not observe an association between the absolute concentration and subsequent development of RILT, at any time point (Table 3A). The mean CCL18 levels at three and six months were in the RILT-group 94±62 ng/ml and 104±61 ng/ml and in the non-RILD-group 93±54 ng/ml and 103±39 ng/ml. Of the three patients who developed a grade 2 pneumonitis, one patient developed a RILT 3 months after treatment at a serum concentration of 54 ng/ml and two developed clinical signs of pneumonitis at the second follow-up and had a serum concentration of 138ng/ml and 134 ng/ml, respectively. All patients were treated adjuvantly for pN2 NSCLC (SCC = 2, adenocarcinoma = 1).

**Table 3:** Univariate analysis of CCL 18—Related variables predictive of radiation pneumonitis Grade ≥ 1.

	Nr.	UVA	
		OR (95% CI)	Adj P value*
**A. Absolute serum concentrations of CCL18**			
CCL18 1^st^ time point*		1.002 (0.896–1.120)	1.0
CCL18 2^nd^ time point		1.010 (0.938–1.086)	1.0
CCL18 3^nd^ time point		1.043 (0.953–1.142)	1.0
CCL18 4^nd^ time point		0.975 (0.883–1.077)	1.0
CCL18 5^nd^ time point		1.022 (0.937–1.115)	1.0
**B.dCCL18 compared to baseline**			
CCL18 2^nd^ vs 1^st^		1.007 (0.927–1.093)	1.0
CCL18 3^nd^ vs 1^st^		0.968 (0.847–1.107)	1.0
CCL18 4^rd^ vs 1^st^		1.010 (0.891–1.145)	1.0
CCL18 5^th^ vs 1^st^		0.948 (0.803–1.120)	1.0
**C. Classification and summary measures of individual courses**			
**Over 2 time points**			
20% decrease	18	0.308 (0.072–1.315)	
20% increase	13	0.198 (0.049–0.801)	
Stable (Reference)	22		0.52
**Over 3 time point**			
20% decrease	18	0.286 (0.059–1.395)	
20% increase	13	0.444 (0.087–2.276)	
Stable then 20% decrease	10	2.333 (0.400–13.609)	
Stable (Reference)	12		0.79
**Slope**		0.975 (0.797–1.080)	1.0

Abbreviations: OR = odds ratio for a 10ng/ml change of the CCL18 concentration, CI: confidence interval, * = baseline, SD = Standard deviation, Nr: Number of patients, UVA: univariate analysis.

*Bonferroni-adjusted p-value. The p-values were adjusted by multiplication with 12 (reflecting that we assessed significance for 12 different, potential associations).

#### Changes during and after treatment

Furthermore we correlated the changes of CCL18 from baseline with subsequent development of RILT. No associations could be observed (Table 3B). When classifying the individual courses, we found at the 2^nd^ follow up 23 patients with initially rather stable CCL18 concentrations remained stable, whereas 18 decreased and 13 increased more than 20% from baseline. We couldn´t observe any associations between the different CCL18 concentrations and a higher risk to develop RILT (Table 3C).Also when classifying the course over the first 3 time points, no differences could be observed (Table 3C). The slope did not correlate. The effect of did not change when adjusting for tumors that did not progress within the first 6 months (n = 39) in order to exclude any possible interference of CCL18 production due tumor progression.

When considering Cox proportional hazard models for the time until RILT, we could not find any significant differences.

### CCL18 as a marker of development of RILT

Nine patients developed signs of RILT between treatment completion and first follow up and 12 patients between first and second follow up. Patients with RILT at first follow up had a mean CCL18 concentration of 94 ±62 ng/ml whereas patients without RILT had a mean CCL18 concentration of 93 ±54 ng/ml. These results did not vary significantly in the second follow up (104 ±61 ng/ml vs 103 ±39 ng/ml for patients with and without RILT respectively). Post development of RILT, CCL18 values were on average increased by 6.8ng/ml compared to pre development of RILT. This difference was not statistically significant.

### Prognostic value of CCL18 for treatment response

At the second follow-up six patients had died, all due to progression, two of whom in the first 3 months (OS at 6 months 89%) and fourteen patient developed a local progression, two of whom during the first follow-up (LC at 6 months 74%). Neither the absolute CCL18 concentrations nor the changes from baseline (dCCL18) correlated with the OS and LC at any specific time point (S2 Table).

## Discussion

In patients with lung fibrosis, increases in pulmonary levers of CCL 18 occur in association with fibrosis [10]. It has been reported that in the course of idiopathic pulmonary fibrosis, alveolar macrophages shift from their pro-inflammatory, classical activation (M1 type) to alternative activation, the M2 phenotype that promotes collagen production, scar formation and angiogenesis. [7] This M2 state is induced and maintained by products of activated fibroblasts [11]. CCL18 is abundantly produced by alternatively activated macrophages in patients with idiopathic pulmonary fibrosis and its concentrations reflect the fibrotic lung activity in patients with idiopathic interstitial pneumonias, systemic sclerosis and idiopathic pulmonary fibrosis [10, 12]. That led us to the hypothesis that serum concentrations of CCL18 or their fluctuations during therapy could be also a potential marker for predicting RILT, as radiation pneumonitis and the ensuing pulmonary fibrosis is associated with a fibrotic remodeling of lung tissue. [5, 25]

To our knowledge this is the first study that prospectively investigates the role of the CC chemokine ligand 18 as a biomarker for radiation pneumonitis. We did not find any correlation between increasing CCL18 concentrations and risk of RILT as initially hypothesized.

Alternatively activated macrophages are found in fibrotic diseases [13, 26] as well as in tumors as so called tumor-associated macrophages [27]. Hence, in fibrotic diseases [6] as well as in various cancers [22, 23, 28] CCL18 levels are increased. As CCL18 serum levels correlate with tumor size [22, 29] we expect that CCL18 levels decrease after response to radiotherapy. Increase of CCL18 released by alternatively activated macrophages in a pro-fibrotic environment might therefore mask this decrease, resulting to “false” stable concentrations due to two different pathomechanisms (CCL18 decrease due to tumor regression and increase due to RILT). Some researches for potential biomarkers for RILT suggested that the cytokines tested could correlate more with tumor activity than with the incidence of lung injury. [30, 31]. Indeed it is known that a number of cytokines that are produced in the tumor microenvironment have an important role in cancer pathogenesis.[15] Cytokines that are released in response to infection, inflammation and immunity can function to inhibit tumor development and progression [14] [32]. Alternatively, cancer cells can respond to host-derived cytokines that promote growth, attenuate apoptosis and facilitate invasion and metastasis.[14]. In our study we could not observe any effect of the different CCL18 concentrations also after adjusting for progression within the first 6 months after treatment completion, suggesting that these results did not interfere with the tumor activity.

Furthermore, we could not demonstrate that elevated or rising CCL18 serum concentrations correspond to tumor activity in terms of progression or worse survival, although the aim of this study was prognostic role of CCL18 concerning RILT. However, these effects might be masked by the heterogeneity in respect to tumor entities of our study cohort. In previous studies it could be demonstrated that CCL18 enhances invasiveness [28] and malignancy [18] and promotes angiogenesis [17] in breast cancer tissue. CCL18 is also overexpressed in gastric cancer cells and contributed to cell invasion and expression [19] while Leung et al. [23] identified that high CCL18 expression levels were associated with prolonged OS and PFS in gastric cancer patients. It has also been reported that it is a favorable prognostic biomarker in patients with colorectal cancer [21] and significantly elevated in ascetic fluid from patients with ovarian carcinoma [33]. Plönes et al [22] reported that serum levels are increased in patients with NSCLC and that its concentrations correlate with survival time in adenocarcinomas.

A number of limitations applying to our study need to be considered, including the small sample size tested, as only 53 patients were evaluable for the analysis, as well as the very low incidence of clinically significant RILT (Grade ≧2) which led us to use Grade ≥ 1 as main outcome, providing only 21 events. We were also confronted with the challenge of the relation between CCL18 values and their course over time and the occurrence of RILT. This led us to check several different aspects of the course for a significant association with the development of RILT, inducing a risk of false significant results. Furthermore, our study was limited by the inhomogeneity of histologies and treatment regimens as it was primarily designed to identify the role of CCL18 as biomarker for predicting RILT. The heterogeneity of our study group also limits conclusions on the prognostic value of CCL18 for treatment response as only for adenocarcinoma a correlation between CCL18 and OS could be demonstrated [22].

Several cytokines have been proposed as potential biomarkers in the past with conflicting results but none of them is being used in the clinical praxis. To that concern, further progress toward an improved understanding of the mechanisms of RILT and the regulation of CCL18 is important for the identification of appropriate targets for prediction and treatment intervention in the clinical practice.

## Conclusion

In this prospective study the dosimetric parameters remain the most potent predictors regardless their limitations. We could not demonstrate an association of different CCL18 concentrations with the development of RILT. Further studies are needed to improve the understanding of the mechanisms of RILT and complex regulation of CCL18 in pulmonary inflammation and fibrosis.

## Supporting information

S1 Table CCL18 concentrations (ng/ml) at every time point. (DOCX)

S2 Table Univariate analysis of CCL 18—Related variables regarding overall survival and local control. (DOCX)
