# Correction: Is serum level of CC chemokine ligand 18 a biomarker for the prediction of radiation induced lung toxicity (RILT)?

**DOI:** 10.1371/journal.pone.0192058

**Published:** 2018-01-25

**Authors:** Eleni Gkika, Werner Vach, Sonja Adebahr, Tanja Schimek-Jasch, Anton Brenner, Thomas Baptist Brunner, Klaus Kaier, Antje Prasse, Joachim Müller-Quernheim, Anca-Ligia Grosu, Gernot Zissel, Ursula Nestle

The fourth author’s name is spelled incorrectly. The correct name is: Tanja Schimek-Jasch. The correct citation is as follows: Gkika E, Vach W, Adebahr S, Schimek-Jasch T, Brenner A, Brunner TB, et al. (2017) Is serum level of CC chemokine ligand 18 a biomarker for the prediction of radiation induced lung toxicity (RILT)? PLoS ONE 12(9): e0185350. https://doi.org/10.1371/journal.pone.0185350
