# Supplementary material for: Is serum level of CC chemokine ligand 18 a biomarker for the prediction of radiation induced lung toxicity (RILT)?
Source: PLoS One. 2017 Sep 28;12(9):e0185350. doi: 10.1371/journal.pone.0185350 (PMC5619767; doi:10.1371/journal.pone.0185350)
Supplement: S1 Table — (DOCX) [file pone.0185350.s001.docx]

| CCL18_1st  Time-point | CCL18_2nd  time-point | CCL18_3rd  time-point | CCL18_4th  Time-point | CCL18_5th time-point |
| --- | --- | --- | --- | --- |
| 115 | 103 |  | 118 |  |
| 79 | 66 | 52 | 56 | 59 |
| 86 | 103 |  | 81 | 89 |
| 112 | 110 | 68 | 75 | 107 |
| 50 | 50 | 18 | 27 | 49 |
| 222 | 173 | 95 | 60 | 75 |
| 90 | 75 | 28 | 50 | 61 |
| 80 | 99 | 72 |  | 95 |
| 150 | 97 | 72 | 131 |  |
| 137 | 122 |  | 121 | 125 |
| 65 | 47 | 31 | 42 | 89 |
| 89 | 144 |  | 70 |  |
| 67 | 45 | 29 | 144 | 46 |
| 29 | 46 |  | 33 | 65 |
| 41 | 90 |  | 70 | 101 |
| 62 | 62 | 48 | 38 | 53 |
| 135 | 88 |  | 143 | 101 |
| 108 | 107 | 118 | 107 |  |
| 123 | 150 | 184 | 130 |  |
| 113 | 92 | 90 | 89 | 84 |
| 145 | 130 |  | 174 |  |
| 234 | 166 |  | 145 | 102 |
| 181 | 276 | 402 | 303 |  |
| 64 | 56 | 50 | 59 |  |
| 161 | 138 |  | 126 |  |
| 55 | 65 |  | 54 |  |
| 114 | 112 |  | 125 | 164 |
| 101 | 92 | 66 | 70 |  |
| 274 | 241 |  | 269 | 272 |
| 188 | 111 |  | 100 | 197 |
| 123 | 463 |  | 26 |  |
| 98 | 64 |  | 71 | 138 |
| 64 | 40 | 37 | 106 |  |
| 45 | 56 |  | 40 |  |
| 44 | 6 | 35 | 49 |  |
| 53 | 103 |  | 45 | 64 |
| 106 | 122 |  | 109 | 98 |
| 124 | 158 |  | 127 | 145 |
| 89 | 81 | 90 | 69 | 134 |
| 116 | 138 |  | 42 |  |
| 145 | 52 |  | 68 |  |
| 73 | 286 | 134 | 104 | 85 |
| 96 | 76 | 72 | 82 | 127 |
| 85 | 56 |  | 95 |  |
| 168 | 173 |  |  | 175 |
| 107 | 71 | 79 | 82 | 67 |
| 77 | 70 |  | 69 | 85 |
| 94 | 76 | 46 | 81 | 78 |
| 100 | 70 | 74 | 87 | 73 |
| 79 | 52 | 31 | 35 | 61 |
| 48 | 26 |  | 27 |  |
| 108 | 125 |  | 212 |  |
| 151 | 48 |  | 126 | 152 |

**S1 Table: CCL18 concentrations (ng/ml) at every time point**
