# Supplementary material for: Is serum level of CC chemokine ligand 18 a biomarker for the prediction of radiation induced lung toxicity (RILT)?
Source: PLoS One. 2017 Sep 28;12(9):e0185350. doi: 10.1371/journal.pone.0185350 (PMC5619767; doi:10.1371/journal.pone.0185350)
Supplement: S2 Table — (DOCX) [file pone.0185350.s002.docx]

**S2 Table: Univariate analysis of CCL 18 – related variables regarding overall survival and local control.**

|  |  | **OS** | | **LC** | |
| --- | --- | --- | --- | --- | --- |
|  | **Nr.** | **OR (95% CI)** | **Adj-P value*** |  | **Adj-P value*** |
| **A. Absolute serum concentrations of CCL18** | | | | | |
| CCL18 1^st^ time point* |  | 1.019 (0.864-1.203) | 1.0 | 0.984 (0.868-1.116) | 1.0 |
| CCL18 2^nd^ time point |  | 0.973 (0.846-1.119) | 1.0 | 1.004 (0.926-1.088) | 1.0 |
| CCL18 3^nd^ time point |  | 0.912 (0.715-1.163) | 1.0 | 0.814 (0.647-1.024) | 0.948 |
| CCL18 4^nd^ time point |  | 0.928 (0.769-1.120) | 1.0 | 1.007 (0.906-1.118) | 1.0 |
| CCL18 5^nd^ time point |  | 0.931 (0.794-1.095) | 1.0 | 0.900 (0.799-1.014) | 1.0 |
| **B.dCCL18 compared to baseline** | | | | | |
| CCL18 2^nd^ vs 1^st^ |  | 0.995 (0.977-1.014) | 1.0 | 1.001 (0.993-1.010) | 1.0 |
| CCL18 3^nd^ vs 1^st^ |  | 0.994 (0.963-1.026) | 1.0 | 0.995 (0.973-1.018) | 1.0 |
| CCL18 4^rd^ vs 1^st^ |  | 0.988 (0.969-1.006) | 1.0 | 1.000 (0.987-1.013) | 1.0 |
| CCL18 5^th^ vs 1^st^ |  | 1.000 (0.967-1.034) | 1.0 | 1.015 (0.980-1.051) | 1.0 |
| **C. Classification and summary measures of individual courses** | | | | | |
| **Over 2 time points** |  |  |  |  |  |
| 20% decrease | 18 | 0.375 (0.037-3.777) |  | 0.533 (0.090-3.178) |  |
| 20% increase | 13 | 0.265 (0.027-2.612) |  | 1.333 (0.343-5.178) |  |
| Stable (Reference) | 22 |  | 1.0 |  | 1.0 |
| **Over 3 time point** |  |  |  |  |  |
| 20% decrease | 18 | 0.417 (0.033-5.299) |  | 0.280 (0.042-1.878) |  |
| 20% increase | 13 | 0.294 (0.024-3.671) |  | 0.700 (0.155-3.166) |  |
| Stable then 20% decrease | 10 | 1.250 (0.143-10.940) |  | 0.156 (0.015-1.653) |  |
| Stable (Reference) | 12 |  | 1.0 |  | 1.0 |

*Abbreviations*

*OR=odds ratio for a 10ng/ml change of the CCL18 concentration, CI: confidence interval, * =baseline, SD= Standard deviation, Nr: Number of patients, OS: overall survival, LC: local control*

**Bonferroni-adjusted p-value. The p-values were adjusted by multiplication with 12 (reflecting that we assessed*

*significance for 12 different, potential associations)*
